# Role of *TP53* mutations in triple negative and HER2-positive breast cancer treated with neoadjuvant anthracycline/taxane-based chemotherapy

**DOI:** 10.18632/oncotarget.11891

**Published:** 2016-09-07

**Authors:** Silvia Darb-Esfahani, Carsten Denkert, Albrecht Stenzinger, Christoph Salat, Bruno Sinn, Christian Schem, Volker Endris, Peter Klare, Wolfgang Schmitt, Jens-Uwe Blohmer, Wilko Weichert, Markus Möbs, Hans Tesch, Sherko Kümmel, Peter Sinn, Christian Jackisch, Manfred Dietel, Toralf Reimer, Sherene Loi, Michael Untch, Gunter von Minckwitz, Valentina Nekljudova, Sibylle Loibl

**Affiliations:** ^1^ Institute of Pathology, Charité Universitätsmedizin Berlin, Berlin, Germany; ^2^ German Cancer Consortium, (DKTK), Berlin, Germany; ^3^ Institute of Pathology, University Hospital Heidelberg, Heidelberg, Germany; ^4^ Department of Pathology, Center for Integrated Diagnostics (CID), Massachusetts General Hospital, Boston, MA, USA; ^5^ Hämatoonkologische Schwerpunktpraxis, Munich, Germany; ^6^ Department of Gynecology and Obstetrics, University Hospital Schleswig-Hostein, Kiel, Germany; ^7^ Praxisklinik Krebsheilkunde für Frauen/Brustzentrum, Berlin, Germany; ^8^ Department of Gynecology and Obstetrics, Charité Universitätsmedizin Berlin, Berlin, Germany; ^9^ Institute of Pathology, Technical University Munich, Munich, Germany; ^10^ Center for Hematology and Oncology Bethanien, Frankfurt/Main, Germany; ^11^ Breast Unit, Kliniken Essen Mitte, Essen, Germany; ^12^ Department of Gynecology and Obstetrics, Sana Klinikum Offenbach, Offenbach, Germany; ^13^ Department of Gynecology, Klinikum Südstadt Rostock, Rostock, Germany; ^14^ Peter MacCallum Cancer Centre, East Melbourne, Victoria, Australia; ^15^ Department of Gynecology and Obstetrics, Helios Klinikum Berlin-Buch, Berlin, Germany; ^16^ German Breast Group c/o (GBG Forschungs GmbH), Neu-Isenburg, Germany

**Keywords:** TP53, mutation, triple negative breast cancer, HER2, pathological complete response

## Abstract

**Background:**

*TP53* mutations are frequent in breast cancer, however their clinical relevance in terms of response to chemotherapy is controversial.

**Methods:**

450 pre-therapeutic, formalin-fixed, paraffin-embedded core biopsies from the phase II neoadjuvant GeparSixto trial that included HER2-positive and triple negative breast cancer (TNBC) were subjected to Sanger sequencing of exons 5-8 of the *TP53* gene. *TP53* status was correlated to response to neoadjuvant anthracycline/taxane-based chemotherapy with or without carboplatin and trastuzumab/lapatinib in HER2-positive and bevacizumab in TNBC. p53 protein expression was evaluated by immunohistochemistry in the TNBC subgroup.

**Results:**

Of 450 breast cancer samples 297 (66.0%) were *TP53* mutant. Mutations were significantly more frequent in TNBC (74.8%) compared to HER2-positive cancers (55.4%, *P* < 0.0001). Neither mutations nor different mutation types and effects were associated with pCR neither in the whole study group nor in molecular subtypes (*P* > 0.05 each). Missense mutations tended to be associated with a better survival compared to all other types of mutations in TNBC (*P* = 0.093) and in HER2-positive cancers (*P* = 0.071). In TNBC, missense mutations were also linked to higher numbers of tumor-infiltrating lymphocytes (TILs, *P* = 0.028). p53 protein overexpression was also linked with imporved survival (*P* = 0.019).

**Conclusions:**

Our study confirms high *TP53* mutation rates in TNBC and HER2-positive breast cancer. Mutations did not predict the response to an intense neoadjuvant chemotherapy in these two molecular breast cancer subtypes.

## INTRODUCTION

*TP53* is the prototype of a tumor-suppressor gene and *TP53* mutations emerged as a core component of cancer development since its discovery in 1979 [1]. It is by far the most frequently mutated gene in human cancer with varying mutation rates across entities and in entity subtypes [2, 3]. While for example in ovarian serous high-grade carcinoma mutation rates approach 100% [4], they are low (<5%) in leukemias, sarcomas or cervical cancer [2]. In breast cancer, *TP53* mutations have been shown to be most prevalent in the poor-prognosis basal-like or triple negative breast cancer (TNBC) subtype (80%), followed by HER2-positive cancers (72%), while mutation rates in the lower proliferative, less aggressive luminal carcinomas are rather low (12-29%) [5]. Mutations result either in the loss of function of *TP53* as the central regulator of proliferation, apoptosis, as well as maintenance of genomic stability [1, 2], or in a gain-of-function that may contribute to tumor progression by conferring new oncogenic functions to the p53 protein resulting in enhanced proliferation, metastasis, and drug-resistance [6].

*TP53* mutations in TNBC and HER2-positive cancers arise in a frequency that suggests that there could be an relevant connection between *TP53* status and therapy response. However, available data on the predictive relevance of *TP53* status are conflicting. Some groups reported *TP53* status to be associated with improved response to chemotherapy [7], while others could not confirm this observation [8]. Variability of the composition of the study cohorts and the applied chemotherapy regimens, as well as use of different methods to determine *TP53* mutations makes the interpretation of current data on this topic difficult.

Based on these considerations, we aimed to determine whether mutational status of *TP53* is predictive for pathological complete response (pCR) in the two molecular breast cancer subtypes with the highest *TP53* mutations rates, namely TNBC and HER2-positive disease. We analyzed prospectively collected pre-therapeutic core biopsies from the phase II randomized neoadjuvant GeparSixto trial that investigated intense anthracycline/taxane-based chemotherapy with or without carboplatin together with targeted agents (bevacizumab in TNBC or trastuzumab/lapatinib in HER2-positive disease) [9].

## RESULTS

### Distribution of *TP53* mutations in TNBC and HER2-positive carcinomas

**Figure 1:**
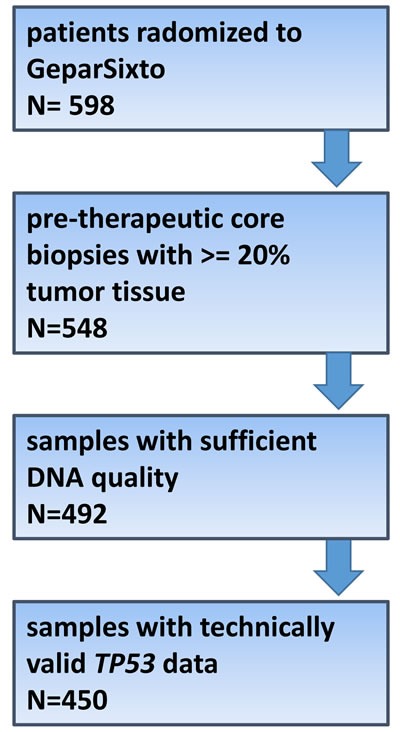
Consort diagram

**Figure 2:**
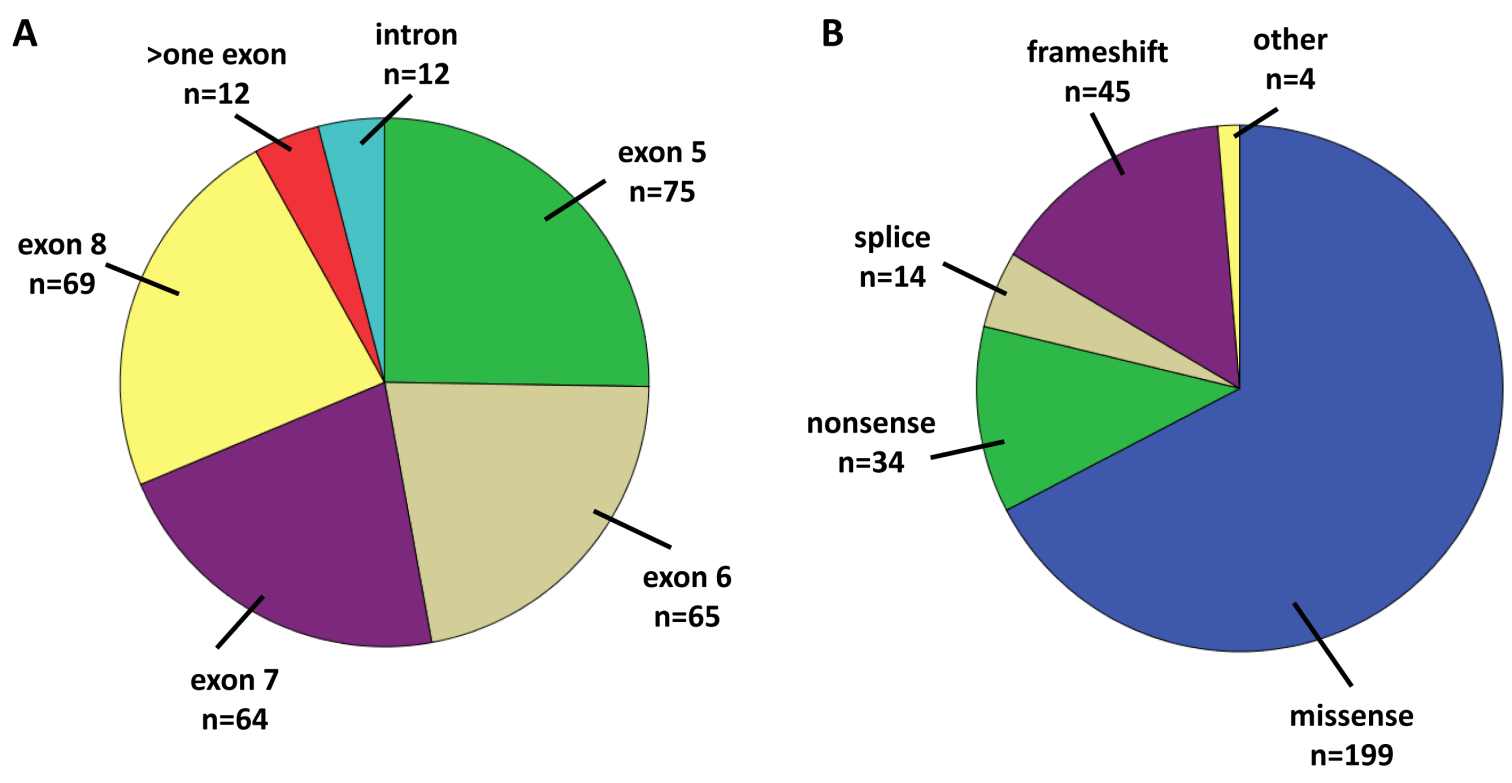
**A.** Distribution of mutations among exons. **B.** Distribution of mutation effects

Of 598 available core biopsies, 548 passed histological QC, and after exclusion of samples with insufficient DNA quality or technically insufficient sequencing results informative data were available from 450 patients randomized to the GeparSixto study (75.2%, Figure 1). Based on central ER/PR/HER2 determination, 246 carcinomas were TNBC (54.7%) and 204 were HER2-positive (45.3%). 231 patients were subjected to a Carboplatin-containing chemotherapeutic regime (51.3%), and 219 received anthracycline/taxane-based neoadjuvant chemotherapy (48.7%). pCR rate (ypT0 ypN0) in the total study group was 38% (*n* = 178). The distribution of clinico-pathological features is given in Table 1. 297 tumors in the total study group harbored a non-synonymous mutation and were classified as “mutant” (66.0%). Of the remaining 153 tumors classified as “wildtype”, 20 had a silent (synonymous) mutation. Mutations were evenly distributed among exons 5-8 (mutations rates 21.5%-25.3%), and 12 tumors (4% of mutated tumors) had mutations in more than one exon. Missense mutations were predominant (*n* = 199, 67.2%), followed by frameshift (15.2%) and nonsense mutations (11.5%). Most mutations were predicted to be deleterious (96.5%). In 14 tumors (3.1%), splice-site disrupting mutations in introns covered by our sequencing approach were detected and were also classified as “mutated”. The distribution of mutation types and effects is shown in Table 2 and Figure 2.

**Table 1:** Characteristics of the study group

characteristic	GeparSixto, p53 study cohort *n* (%)
**no. of samples**	450 (100%)
**age group**	
< 50 years	265 (58.9%)
≥ 50 years	158 (41.1%)
**histological type**	
ductal/other	443 (98.4%)
lobular	7 (1.6%)
**tumor grade**	
G1-G2	154 (34.2%)
G3	296 (65.8%)
**ER/PR status (central IHC)**	
ER-/PR-	327 (72.7%)
ER+ and/or PR+	123 /27.3)
receptor status combined (central IHC/SISH)	
HER2- & ER/PR- (=TNBC cohort)	246 (54.7%)
HER2+ (=HER2+ cohort)	204 (45.3%)
HER2+ & ER/PR-	81 (39.7%)
HER2+ & ER+ and/or PR+	123 (60.3)
**clinical tumor stage**	
cT1-2	383 (85.3%)
cT3-4	66 (14.7%)
*missing*	1
**clinical nodal status**	
cN0	254 (57.6%)
cN+	187 (42.4%)
*missing*	9
**type of chemotherapy**	
with carboplatin (PM+Cb)	231 (51.3%)
without carboplatin (PM)	219 (48.7%)
**pathological complete response (ypT0ypN0)**	
no pCR	279 (62.0%)
pCR	178 (38.0%)

**Table 2:** Type of detected mutations

	*n* (%)
**p53 status**	
wildtype	153 (34.0%)
silent	20 (13.1% of wt)
mutated	297 (66.0%)
exon 5	75 (25.3% of mt)
exon 6	65 (21.9% of mt)
exon 7	64 (21.5% of mt)
exon 8	69 (23.2% of mt)
> one exon	12 (4.0% of mt)
intron	12 (4.0% of mt)
**mutation effect**	
missense	199 (67.2%)
nonsense	34 (11.5%)
frameshift	45 (15.2%)
slice site disruption	14 (4.7%)
other (in frame deletions/insertions)	4 (1.4%)
**transactivation class**	
non-functional	175 (87.9%)
functional	9 (4.5%)
partially functional	14 (7.0%)
supertrans	1 (0.5%)
*not applicable*	*46*
*no data*	*52*
**effect on protein sequence**	
deleterious	274 (96.5%)
neutral	10 (3.5%)
*no data*	*13*
**residue function**	
DNA-binding	43 (14.5%)
buried	139 (46.8%)
exposed	9 (3.0%)
partially exposed	16 (5.4%)
Zn binding	19 (6.4%)
*not applicable*	*18*
*no data*	*53*

### Association of *TP53* mutations with molecular tumor type and clinico-pathological factors

**Figure 3:**
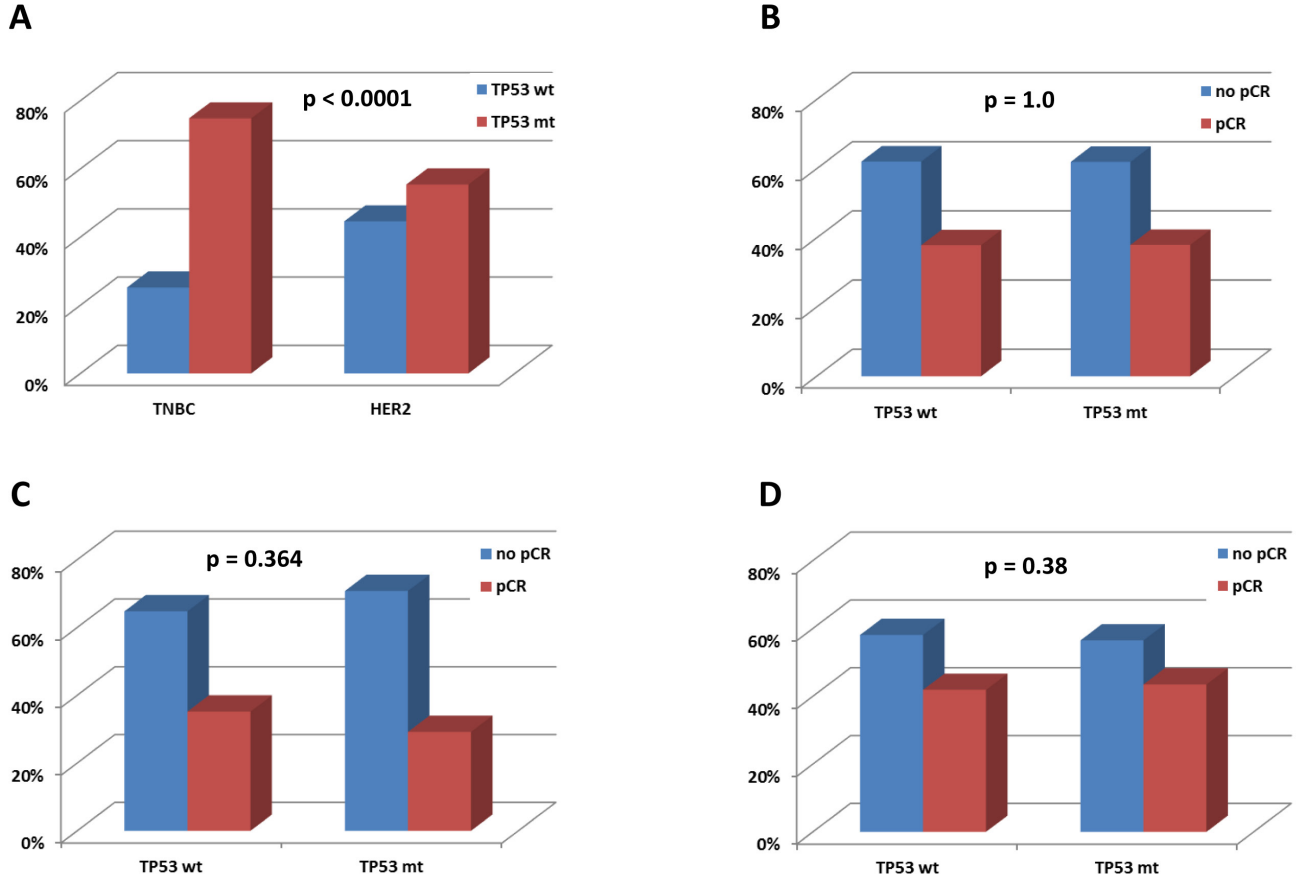
**A.** Associations of mutations with molecular tumor type. p value: chi square **B.** Association with pCR in the total study group. **C.** Association with pCR in the HER2-positive group. **D.** Association with pCR in the TNBC group. B-D) p values: univariate logistic regression

*TP53* mutations were significantly more frequent in TNBC (74.8%) than in HER2-positive carcinomas (55.4%, p<0.0001; Figure 3A). *TP53* mutations were also more frequent in poorly differentiated (G3) carcinomas (*p* = 0.016) but were not associated with age, tumor size (cT), nodal stage (cN), histological subtype, or *PIK3CA* mutational status (*p* > 0.05 each). Within the HER2-positive subtype no association between *TP53* status and hormone receptor expression was seen (*p* = 0.252). In both subtypes there were also no significant links between *TP53* and clinico-pathological factors or *PIK3CA* status (*p* > 0.05 each).

### Link to immunological features of TNBC and HER2-positive breast cancer

*TP53* status in general (mutated *vs* not mutated) was not linked to LPBC subtype or density of TILs determined as a continuous variable, neither in the total study group, nor in TNBC and HER2-positive subtypes (*p* > 0.05 each). However, we found that missense *TP53* mutations were significantly associated with higher levels of stromal TILs in TNBC (*p* = 0.028, suppl. Figure S1A). Interestingly, we did not see significant differences in TILs levels in HER2-positive disease by *TP53* mutation type neither in the total HER2-positive group (*p* = 0.891, suppl. Figure S1B), nor in hormone receptor-positive or hormone-receptor-negative subgroup (not shown). We also studied whether there was a link between *TP53* status and the expression of various immune-related genes assessed in a previous project [10], and the only significant positive association was seen for missense mutations as compared to other mutations and CD8A gene expression in TNBC (*p* = 0.020; not shown).

The increased TILs levels in tumors with p53 missense mutations might be due to p53 protein overexpression and accumulation, which could attract TILs due to elevated neoantigen presentation. To further investigate this hypothesis we determined p53 protein status (n = 185 informative cases, suppl. Figure S2A-C) as well as MHC1 expression, an important component of the antigen-presenting machine (*n* = 194 informative cases; suppl. Figure S2E, F), by IHC. p53 protein expression was significantly associated with *TP53* mutation groups (suppl. Table S1). Protein overexpression was a rather good surrogate marker for *TP53* missense mutations as 80% of p53 overexpressing tumors actually had a missense mutation. Protein loss on the other hand was seen frequently in the “other” mutations group (45.1%), and in cases with *TP53* wildtype genomic status (48.4%). Tumors with a wildtype p53 protein expression pattern were wildtype on the genomic level in only 31.7% of cases. As a reflection of the moderate association between *TP53* genomic and p53 protein status, there was a trend toward higher TILs levels in TNBC with p53 overexpression as compared to cases with protein loss, however, this was not statistically significant (*p* = 0.199, suppl. Figure S1C). p53 protein status was not significantly associated with mRNA immune markers (not shown). High MHC1 expresion was seen in 157 TNBC (80.9%). Confirming its role as a regulator of anti-tumoral immune activiation high MHC1 expression was significantly associated with high TILs (*p* = 0.004, suppl. Figure S1D) and with 7 mRNA-based immune markers (*CCL5, CXCL13, PDL1, CTLA4, FOXP3, IDO1, CD80*; not shown). There was however, no significant association between MHC1 expression and *TP53* genomic or p53 protein status (not shown).

### Impact of *TP53* mutations on response to neoadjuvant chemotherapy

The distribution of *TP53* mutations in the total study group was the same in patients with and without a pCR (*p* = 1.0; Figure 3B). Similarly, no impact of *TP53* status on pCR was seen within the subtypes of TNBC and HER2-positive disease (Figure 3C, 3D). In TNBC, smaller tumor size (cT1-2) and carboplatin-containing chemotherapy were significantly linked to pCR, and in HER2-positive cancers, only negative hormone receptor status was predictive for a pCR (Table 3). Moreover, we investigated associations between pCR in TNBC and HER2-positive tumors** and *TP53* mutational status including the type and effects of *TP53* mutations (affected exon, mutation effect, transactivation class, SIFT class, residue function and gain-of-function), but did not observe a significant impact (*p* > 0.05 each). We also studied the link between *TP53* status and pCR in TNBC and HER2 cancers stratified for the type of chemotherapy (carboplatin-containing *vs* control) as well as clinico-pathological factors (age, grade, cT, cN, hormone receptor status in HER2-positive disease), *PIK3CA* mutations, and LPBC subtype. Again, no significant results were obtained (*p* > 0.05 each). Based on reports on a potential biological relevance of silent *TP53* mutations in breast cancer [11], we exploratorily added the 12 tumors in our cohort harboring a silent mutation to the “mutated” category and tested for associations with pCR, however, with no significant result (*p* > 0.05 each). Using a less stringent pCR definition including tumors with residual ductal carcinoma in situ (ypT0/ypTis), we obtained the same negative results for all analyses. p53 protein status and MHC1 expression in TNBC were also not significantly linked to response to neoadjuvant chemotherapy (not shown).

**Table 3:** Associations with pCR

*TNBC subgroup*						
	*n*	events	% pCR	OR	95% CI	*p*
**p53 status**						
wildtype	62	26	41.9	1	-	
mutated	184	80	43.5	1.07	0.60-1.91	0.83
**age**						
< 50 years	144	64	44.4	1	-	
>= 50 years	102	42	41.1	0.88	0.52-1.46	0.61
**histological type**						
ductal/others	241	106	44.0	1	-	
lobular	5	0	0.0	n.a.	n.a.	0.999
**tumor grade**						
G1-2	63	21	33.3	1	-	
G3	183	85	46.4	1.74	0.95-3.16	0.071
**clinical tumor stage**						
cT1-2	221	101	45.7	1	-	
cT3-4	25	5	20.0	0.30	0.11-0.82	0.019
**clinical nodal status**						
cN0	148	88	59.5	1	-	
cN+	91	24	26.4	0.33	0.19-0.58	0.330
**type of chemotherapy**						
without carboplatin (PM)	120	36	30.0	1	-	
with carboplatin (PM+Cb)	126	70	55.6	2.92	1.73-4.93	<0.0001
***HER2+ subgroup***						
	n	events	% pCR	OR	95% CI	p
**p53 status**						
wildtype	91	32	35.2	1	-	
mutated	113	33	29.2	0.76	0.42.1.37	0.364
**ER/PR status (central IHC)**						
ER-/PR-	81	37	45.7	1	-	
ER+ and/or PR+	123	28	22.8	0.35	0.19-0.64	0.001
**age**						
< 50 years	121	36	29.8	1	-	
>= 50 years	83	29	34.9	1.27	0.67-2.30	0.435
**histological type**						
ductal/others	202	64	31.7	1	-	
lobular	2	1	50.0	0.46	0.03-7.53	0.589
**tumor grade**						
G1-2	91	25	27.5	1	-	
G3	113	40	35.4	1.45	0.79-2.64	0.228
**clinical tumor stage**						
cT1-2	162	50	30.9	1	-	
cT3-4	41	14	34.1	1.16	0.56-2.40	0.686
**clinical nodal status**						
cN0	106	34	32.1	1	-	
cN+	96	30	31.2	0.96	0.53-1.74	0.900
**type of chemotherapy**						
with carboplatin (PM+Cb)	105	30	28.6	1	-	
without carboplatin (PM)	99	35	35.4	0.73	0.41-1.32	0.299

### Association with survival

**Figure 4:**
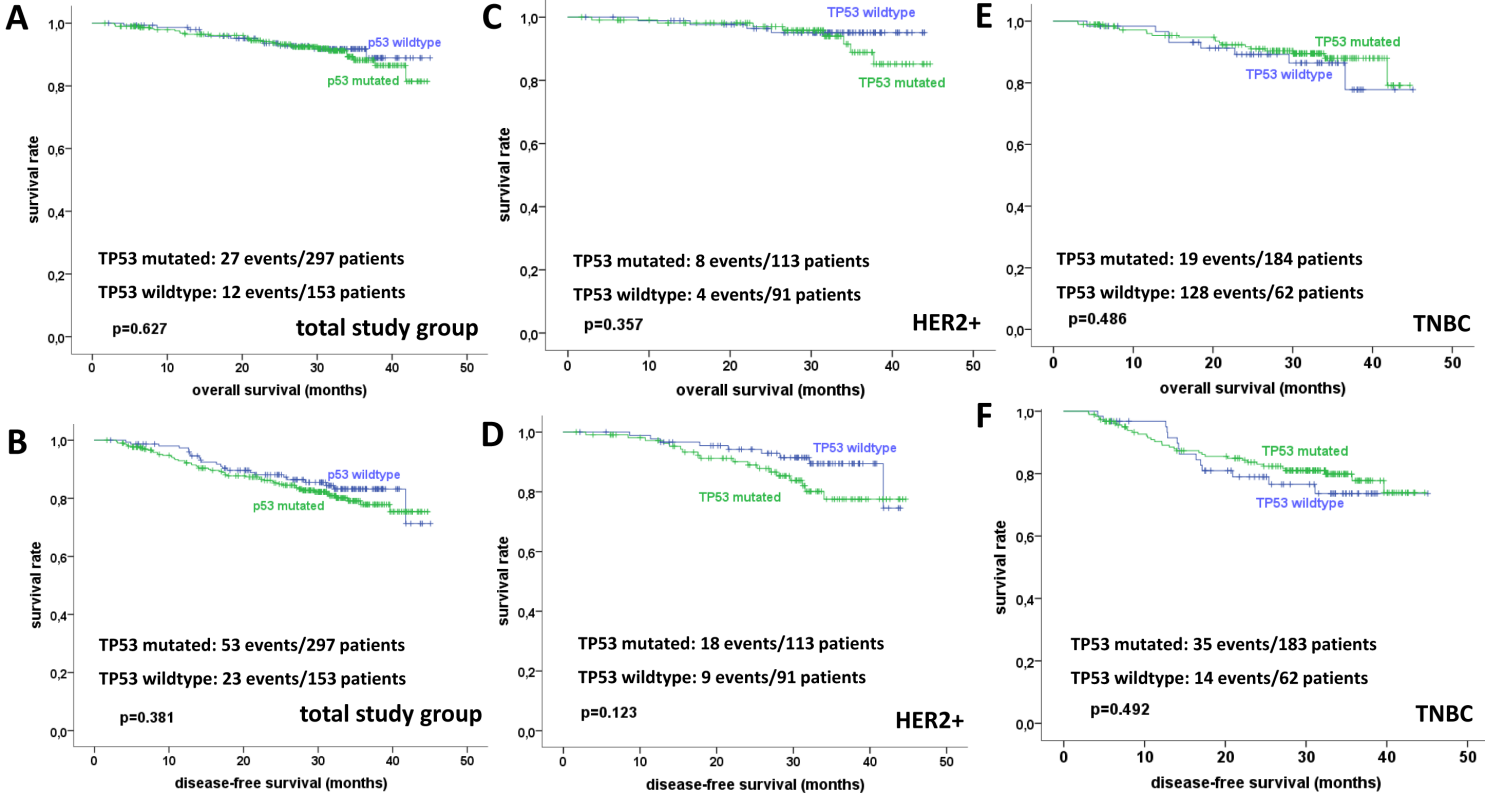
**A.** Distribution of mutations among exons. **B.** Distribution of mutation effects.

*TP53* mutational status was further tested for its impact on patient survival. However, no significant impact was seen according to overall, disease-free, distant disease-free, or local recurrence-free survival, neither in the total study group (Figure 4A, 4B), nor in HER2-positive disease (Figure 4C, 4D) or TNBC (Figure 4E, 4F). Interestingly, there was a trend towards a prognostic effect of mutation types: In TNBC, the missense mutations group showed a better survival than the group with other (non-missense) *TP53* mutations (*p* = 0.093), while the latter group was not significantly different from the wildtype group (*p* = 0.734; suppl. Figure S3A). In HER2-positive disease, a similar trend towards a better survival of the missense group as opposed to tumors with other mutation types was evident (*p* = 0.071), however here, tumors with a wildtype *TP53* status had the best survival, and the difference between the wildtype and the other mutations group was significant (*p* = 0.011; suppl. Figure S1D).

Quite similarly to the relevance of *TP53* genomic status for survival, TNBC with a p53 protein overexpression showed longer DSF as compared to tumors with protein loss (*p* = 0.019) and no difference was seen between tumors with protein loss and a wildtype expression pattern (*p* = 0.365; suppl. Figure S3C). MHC1 expression was no significant prognostic factor (not shown).

## DISCUSSION

We provide a systematic investigation of the clinical relevance of *TP53* mutations in TNBC and HER2-positive breast cancers treated with modern neoadjuvant chemotherapy regimens. Our data show that in these breast cancer subtypes *TP53* status does not have an impact on response to chemotherapy, neither for standard nor for carboplatin-containing regimes.

Available data on the predictive value of *TP53* mutations are conflicting. The phase III prospective EORTC 10994/BIG 1-00 trial comprising 1.486 patients investigated the hypothesis, derived from pre-clinical investigations [12], that specific *TP53* might be associated with resistance to doxorubicin [8]. The results of this translational study are in line with our data as both pCR and complete clinical response to anthracycline or taxane-based neoadjuvant chemotherapy were not impacted by *TP53* status. However, the findings of the group of Bertheau reported that *TP53* might be predictive for chemotherapy response in a particular setting: In a pooled analysis of 144 breast cancers from three series this group found that pCR rates in *TP53* mutant tumors depended strongly on the type of chemotherapy, and were significantly higher after high-dose cyclophosphamide (36%) as opposed to standard-dose (4%) or no cyclophosphamide (12%) [7]. The effect was most pronounced in ER negative tumors, where the pCR rate after high-dose (not lower dose) cyclophosphamide among *TP53* mutant cased reached 71%. 11 of 21 tumors in this subgroup were TNBC. In two previous projects of the same group a similar strong impact of *TP53* status on response to high-dose/dose-dense neoadjuvant cyclophosphamide was seen [13, 14]. However, this analysis was not stratified according to molecular subtypes and GeparSixto did not contain cyclophosphamide, which makes the comparison of Berteau's findings with our data difficult. Taking together the currently available data, it is conceivable that the role of *TP53* mutations in therapy response might be dependent on the chemotherapy regimen and components applied. A potential explanation of the described effect of *TP53* status and response might be that in *TP53* mutant tumors, chemotherapy might induce a higher frequency of genetic defects than in *TP53* wildtype tumors leading to a better response [15]. This might only become evident under dose-dense chemotherapy with a potential particular importance of cyclophosphamide. Furthermore, due to the fact that *TP53* mutations are quite differentially distributed among breast cancer molecular subtypes the results of studies investigating the predictive impact of TP53 status seem to be very much affected by the composition of the study group. Our analysis of TNBC and HER2-positive carcinomas as separate entities however does not reveal an impact of *TP53* status on response.

In a recent report, Carey et al. described that in the CALGB 40601 trial the p53 signature was independently associated with high pCR rates in 305 patients with HER2-positive breast cancer [16]. This p53 signature was based on RNA expression levels of 52 genes associated with *TP53* mutation or loss [17]. While this approach provides a broad read-out of the biological effects of *TP53* mutations also covering any other defect within the p53 signaling cascade it does not directly measure mutant *TP53*. This might explain why in HER2-positive disease, the p53 gene signature was a predictive factor (in CALGB 40601) while *TP53* mutations (in GeparSixto) were not.

There are further previous studies on the predictive effect of *TP53* status in breast cancer that were summarized in a meta-analysis by Chen (2012) [18]. Investigating 26 studies comprising 3.476 cases, the authors concluded that *TP53* aberrations were associated with a higher response to neoadjuvant chemotherapy, particularly for anthracycline-based regimes. However, studies using different methods of *TP53* assessment (IHC and gene sequencing) were pooled and the analysis was not stratified for molecular subtype, so that again the comparison with our data is not straightforward.

In our study *TP53* status in general (mutated *vs* not mutated) was not associated with survival, while previous reports describe it as an unfavorable prognostic factor in unselected breast cancer cohorts. The prognostic effect of *TP53* status however seems to be restricted to the luminal or ER positive subgroup, which has not been included into GeparSixto. This view is supported by the analysis of the METABRIC cohort, which provides high-level evidence of the clinical importance of *TP53* in defined molecular subtypes of breast cancer [19]. Here all exons of *TP53* in 1.420 breast cancers were investigated by Sanger sequencing. *TP53* mutations were associated with worse prognosis in ER positive cancer in general and in luminal B, as well as HER2-enriched cancers (not totally overlapping with HER2-positive disease by IHC/in-situ-hybridization). Interestingly, we found a tendency towards a better survival in patients with *TP53* missense mutations compared to other mutation types. The structural nature of TP53 mutations thus seem to be relevant in term of clinical outcome, an issue which has also been reported by Seagle et al. (2015) who focused on the impact of various structurally-grouped missense mutations in the TCGA ovarian and breast cancer cohorts [20]. It should be noted however, that the significance of our survival analysis is limited because GeparSixto was powered for pCR, not survival and because the current follow-up period is rather short.

One interesting finding of our project was that in TNBC missense mutations as opposed to other mutations were significantly linked to higher numbers of TILs and higher CD8A gene expression levels. It is known that different *TP53* mutations are associated with varying levels of function and protein expression- for example, missense mutations generally have higher protein levels and frameshift or nonsense mutations cause loss of protein [2, 3], and we could reproduce this by applying p53 IHC to our TNBC subcohort, although the correlation between genomic and protein status was not perfect. This can be due to several reasons, e.g. not all missense mutations actually cause protein accumulation, and on the other hand alterations of the protein status, e.g. protein loss can also be due to epigentic or post-translational modifications. Finally, the p53 IHC evaluation using TMAs constructed out of core biopies is limited by the comparably small tumor areas. Particulary the distinction between a wildtype pattern and an overexpression might be difficult in this setting. The moderate correlation between genomic and protein status might explain why the link between p53 protein overexpression and TILs was not significant, however visible in trend. One possible explanation for our findings is that mutations that generally result in increased expression of mutant p53 protein (= missense mutations) as opposed to loss of expression could produce neoantigens as an immune stimulus. This could also explain why *TP53* mutations *per se* were not associated with immune parameters in GeparSixto. The link between missense mutations and an activated immune response might also be related to the effect of missense mutations as well as p53 overexpression on longer survival that we detected in GeparSixto. The strong connection between MHC1 expression and immune activation we observed in the GeparSixto TNBC subcohort argues for the relevance of antigen presentation in the context of an anti-tumoral immune response. Although our findings suggest a role of *TP53* mutations in immune regulation in TNBC, an exhausting elucidation of potential mechanisms underlying this effect, e.g. the analysis of p53-associated neoantigen expression and of respective reactive T cell clones, is not feasible within the scope of this paper, but would be a highly attractive subsequent project. The METABRIC study provided also evidence of a connection of *TP53* aberrations and the immune response in breast cancer: ER negative cancers with wildtype *TP53* and a severe TILs infiltrate had a better prognosis [19]. In a subsequent, more extensive analysis of this topic, the authors found that in basal-like cancers and/or in integrative cluster 10 tumors (basal-like with genomic instablility) *TP53* wild type status was positively associated with T-cell activation and a good prognosis [21]. Small sample sizes in subgroups prohibit a valid reproduction of the METABRIC findings in GeparSixto, however the results of both studies strongly encourage the further investigation of the interaction between the immune response and particular classes of *TP53* mutations in terms of survival in independent cohorts.

Our study has several strengths and weaknesses. In our analysis of *TP53* we focused on the mutational hotspots in exons 5-8, where approximately 80% of reported *TP53* mutations are found [2]. However, by this approach we may have missed mutations in other exons. Furthermore, while the specificity of Sanger sequencing is high, the method has a lower sensitivity in detecting mutations when compared to novel methods such as next-generation sequencing (NGS). However, we assume that founder mutations such as those in *TP53* are present in the majority of tumor cells, so that allelic frequencies are high enough to be detected by Sanger sequencing. Furthermore, the clinical impact of *TP53* mutations in subclones resulting in rather low allelic frequencies, which can only be detected by NGS but not by Sanger sequencing is doubtful. Matching these considerations, a recent study comparing Sanger with NGS in breast cancer showed that the additional *TP53* mutations detected by NGS had no additional impact on the clinical information that was already provided by Sanger sequencing [22].

Taken together, we conclude from our study that *TP53* mutations have no predictive value in patients treated with an intense anthracycline/taxane/targeted agents-based neoadjuvant chemotherapy with or without Carboplatin within TNBC and HER2-positive tumors. Therefore we cannot confirm a previously proposed impact of *TP53* mutations on chemotherapy response in our large clinical trial. Novel predictive factors for TNBC and HER2-positive disease, particularly for the response to platinum-containing chemotherapy are still needed.

## MATERIALS AND METHODS

### Study population

In GeparSixto (clincaltrials.gov NCT01426880) [9], patients with centrally confirmed HER2-positive breast cancer or TNBC were treated for 18 weeks with paclitaxel 80 mg/m2 once every week and non-pegylated liposomal doxorubicin 20 mg/m2 once every week. Patients were randomly assigned at a 1:1 ratio to receive simultaneously carboplatin at a dose of 1.5 (initially 2.0) area under curve once every week for 18 weeks or not. Patients with TNBC received additional bevacizumab 15mg/kg once every 2 weeks, and patients with HER2-positive disease received additional trastuzumab 6 mg/kg (loading dose 8 mg/kg) once every 3 weeks and lapatinib 1000 (after amendment 750) mg daily simultaneously. Pre-therapeutic formalin-fixed paraffin-embedded core biopsies were collected after written informed consent. Hormone receptor positivity was defined as estrogen (ER) and/or progesterone receptor (PR) expression in at least 1% of tumor cells by central immunohistochemistry (IHC). Ethical approval was obtained for all clinical centers and from the institutional review board of the Charité Berlin. pCR was defined as the absence of residual invasive or non-invasive tumor cells in breast and lymph nodes (ypT0 ypN0). Data on overall, disease-free, distant disease-free, as well as local recurrence-free survival were available with a median survival of 31 months (maximum 45 months). Tumor-infiltrating lymphocytes (TILs) were investigated centrally according to the study protocol as a secondary endpoint. Lymphocyte-predominant breast cancer (LPBC) was defined as a dense lymphocytic infiltration of at least 60% of the tumor stroma [10]. PIK3CA mutations in exon 9 and exon 20 had been assessed by Sanger Sequencing in a previous project [23].

### Determination of TP53 mutational status

Histopathological quality control was performed prior to DNA isolation. Only core biopsies with an invasive tumor area ≥20% were eligible.

DNA was isolated from formalin-fixed and paraffin embedded tissue (FFPE) by automated DNA-extraction via QIAsymphony (Qiagen, Hilden, Germany). Exons 5-8 of the *TP53* gene were amplified using Fidelity-taq polymerase (Affymetrix, Santa Clara, CA, USA) and the following primers: Exon 5-forward: ttt caa ctc tgt ctc ctt cct ctt; Exon 5-reverse: agc cct gtc gtc tct cca g; Exon 6-forward: cag gcc tct gat tcc tca ct; Exon 6-reverse: ctt aac ccc tcc tcc cag ag; Exon 7-forward: ctt ggg cct gtg tta tct cc; Exon 7-reverse: ggg tca gag gca agc aga. Exon 8-forward: gcc tct tgc ttc tct ttt cc; Exon 8-reverse: taa ctg cac cct tgg tct cc. Purification of PCR products was performed by ExoSapit (Affymetrix, Santa Clara, CA, USA).

Direct sequencing of the PCR amplicons was carried out for both strands on an 3500 Genetic Analyzer (Applied Biosystems by Life Technologies Corporation, Darmstadt, Germany) using the BigDye^®^ Terminator v1.1 Cycle Sequencing Kit (Applied Biosystems by Life Technologies Corporation, Darmstadt, Germany).

### Classification of *TP53* variants

Point mutations were uploaded into the IARC P53 Database (version R17, November 2013) [24] and the following parameters were assessed: effect on DNA structure, effect on protein structure and function (transactivation class, SIFT class), potential gain-of-function. Significance of mutations (effect on protein structure and function) that were not listed in the IARC P53 Database (most often frameshift mutations, in-frame indels) was determined by analysis of the affected domain and the secondary structure of the respective protein.

### Immunohistochemistry

p53 as well as MHC1 protein expression was evaluated in the TNBC subgroup, for which a tissue microarray (TMA) constructed from pre-operative punch biopsies was available. A mouse monoclonal antibody directed against p53 protein (clone DO-7; DAKO; Glostrup, Denmark) was used in 1:50 dilution on a Ventana Benchmark autostainer (Ventana, Tucson, AZ, USA). A mouse monoclonal antibody directed against HLA-A/B/C (clone EMRE 8-5; MBL, Woburn, MA, USA) was used in a 1:6.000 dilution on a Ventana Discovery XT autostainer (Ventana). Diaminobenzidine was used as a chromogen. Stained TMA sections were digitized and evaluated on screen by an experienced pathologist (SDE), supported by the VM Slide Explorer 2.2 software (VM Scope GmbH, Berlin, Germany). p53 staining was scored as “wildtype pattern” when tumor cell nuclei showed variable and weak staining intensity,, as “overexpression” when at least 60% of tumor cell nuclei were uniformly strongly or moderately stained, as “loss” when tumor cell nuclei were completely negative but p53 staining was evident in non-neoplastic cells in the tissue core (suppl. Figure S1A-C). For MHC1 expression both staining intensity and percentage of stained tumor cells were determined and combined to an immunoreactivity score (IRS), which has been described before [25]. Cases with negative or low expression (IRS0-3) were separated from highly positive cases (IRS4-12; suppl. Figure S1C-F).

### Statistical evaluation

Statistical analysis was performed using IBM SPSS Statistics 22 (IBM Corporation, Somers, NY, USA). Associations between *TP53* mutations or *TP53* mutation types and effects as well as clinico-pathological parameters, *PIK3CA* mutations and LPBC were investigated with Chi square tests. Associations between *TP53* status and immune gene expression were assessed by the Mann-Whitney test. Impact on survival using the Kaplan-Meier method. Odds ratios (ORs) and 95% confidence intervals with two-sided p values were used. A *p* value < 0.05 was considered statistically significant. Univariable logistic regression for connections of *TP53* status with pCR was performed in the complete cohort as well as separately for the TNBC and the HER2-positve subcohort. For each of the three cohorts, the the analysis was stratified for the following parameters: therapy arm (PM *vs* PMC); age (<50 *vs* > = 50 years); tumor size (cT1-2 *vs* cT3-4); nodal stage (cN0 *vs* cN+); grading (G1-2 *vs* G3); hormone receptor expression groups (HR+ *vs* HR-; HER2+ only); LPBC groups (LPBC *vs* no LPBC); PIK3CA mutation groups (PIK2CAmt *vs* PIK3CAwt).

## SUPPLEMENTARY MATERIALS FIGURES AND TABLE


