# Supplementary material for: Role of TP53 mutations in triple negative and HER2-positive breast cancer treated with neoadjuvant anthracycline/taxane-based chemotherapy
Source: Oncotarget. 2016 Sep 7;7(42):67686–98. doi: 10.18632/oncotarget.11891 (PMC5356512; doi:10.18632/oncotarget.11891)
Supplement: Supplementary file 1 [file oncotarget-07-67686-s001.pdf]

Role of *TP53* mutations in triple negative and HER2-positive breast cancer treated with neoadjuvant anthracycline/taxane-based chemotherapy

Supplementary Material

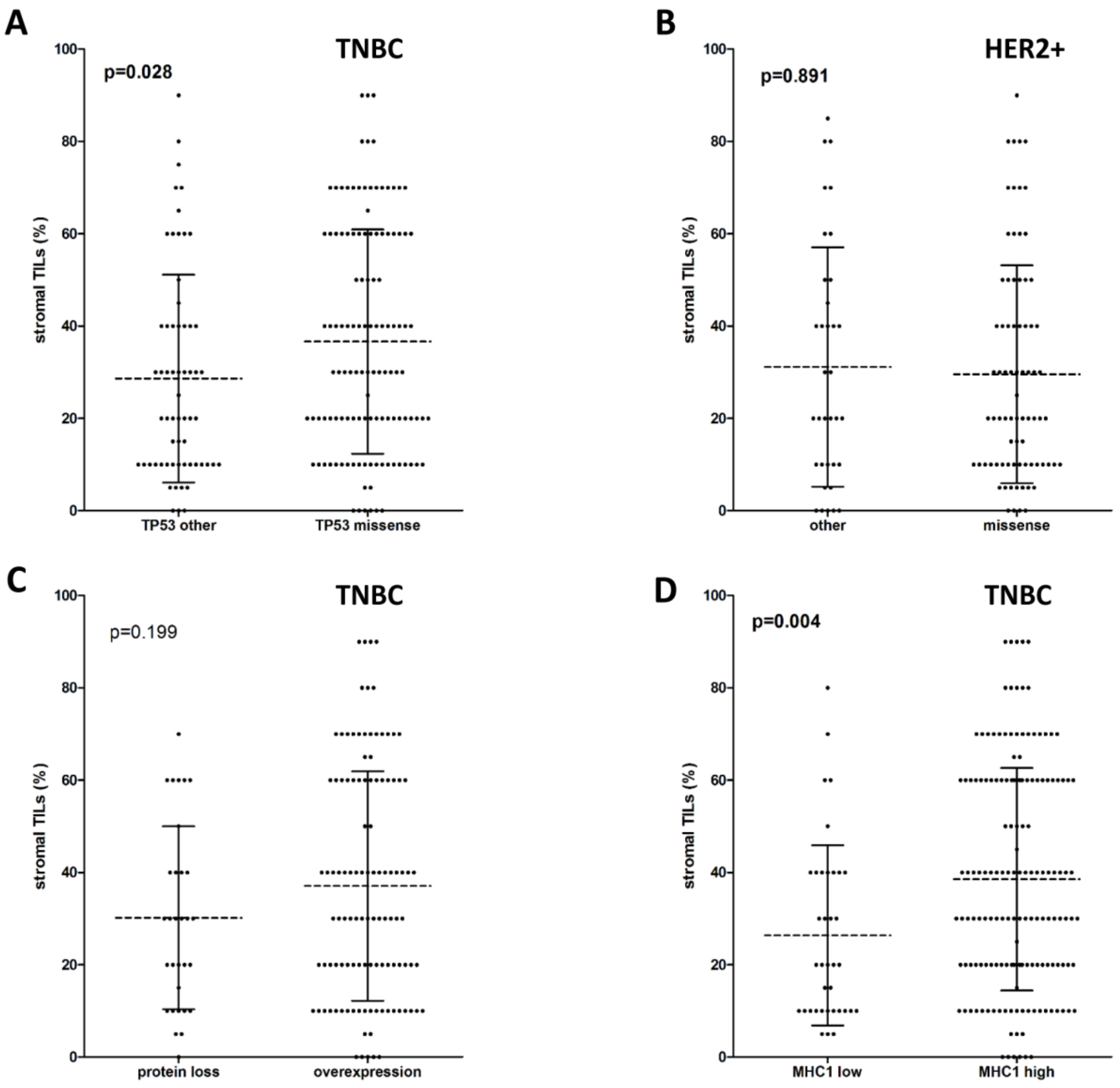

Supplemental Figure S1 Association between mutation type and stromal TILs in TNBC **(A)** and HER2-positive carcinomas **(B)**. Association between aberrant p53 protein expression and TILs in TNBC **(C).** whiskers: standard deviation, dotted line: mean, p-value: Mann-Whitney test.

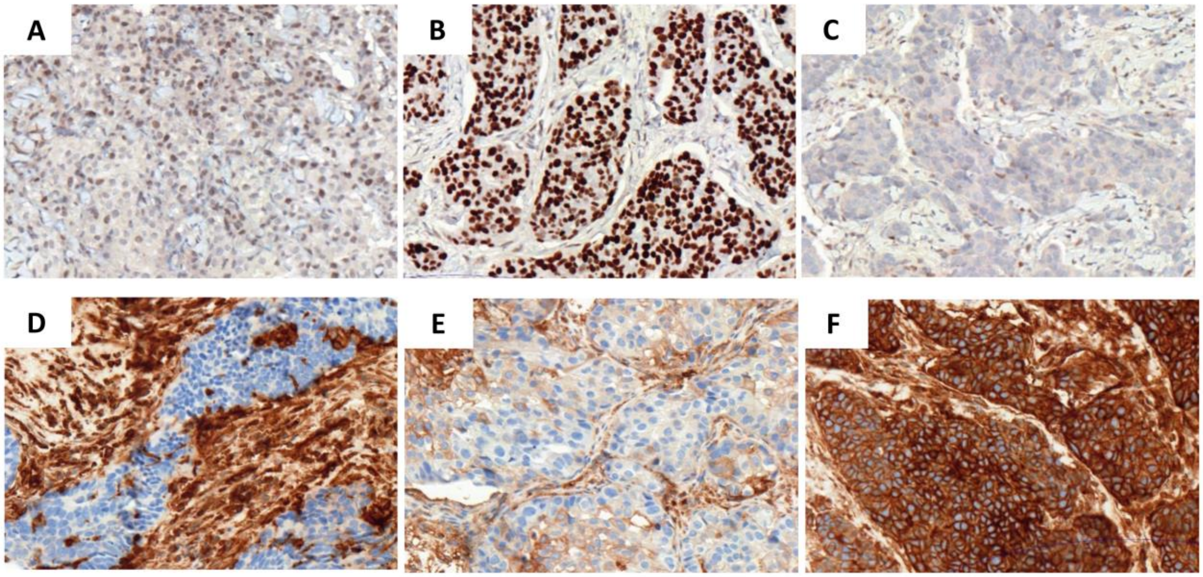

Supplemental Figure S2 Immunohistochemistry. **A** Variable p53 staining of weak to moderate intensity (wildtype pattern) **B** Uniform strong p53 overexpression **C** Lacking nuclear p53 expression in tumor cell nuclei; stromal lymphocytes reveal weak nuclear expression **D** Complete negativity of tumor cells for MHC1, while stromal TILs and macrophages are strongly positive **E** Patchy weak MHC1 expression, scored as low expression **F** Strong diffuse MHC1 expression in tumor cells

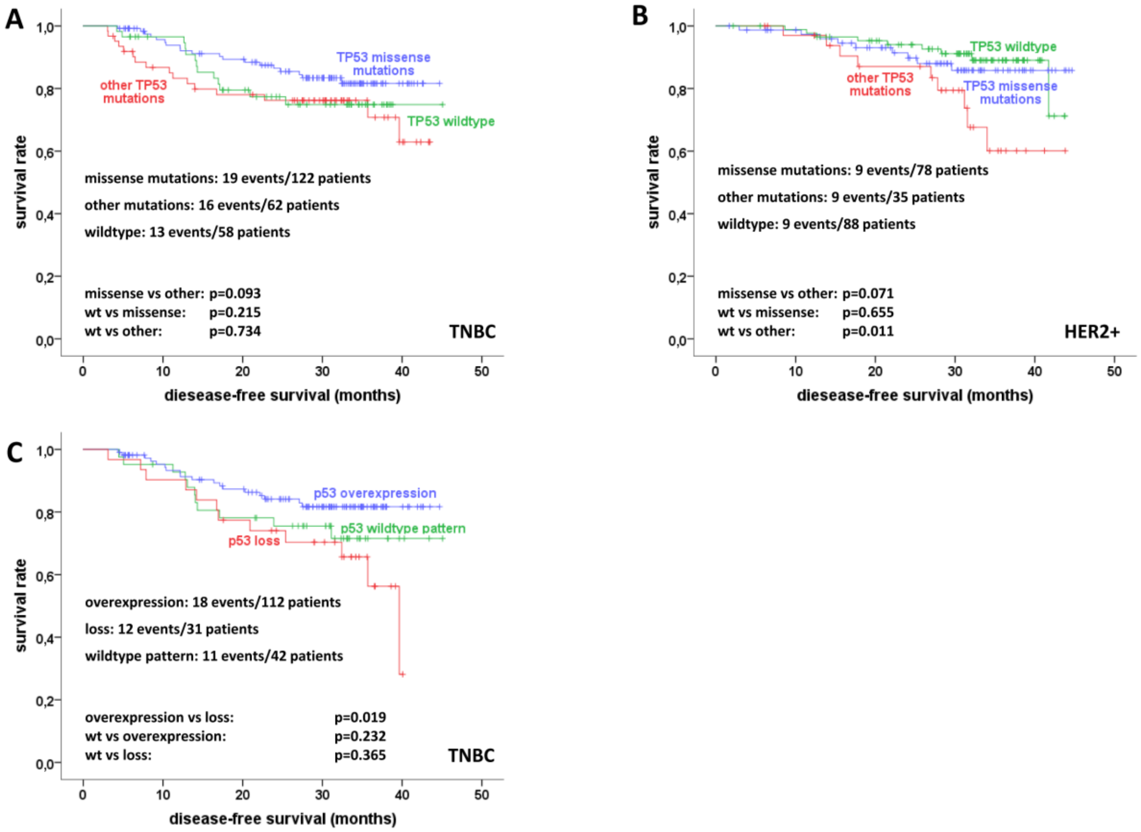

Supplemental Figure S3 DSF according to *TP53* mutation type in TNBC **(A)** and HER2-positive carcinomas **(B)**. DSF according to p53 protein expression in TNBC **(C).** wt: wildtype

Supplemental Table S1 Associations between *TP53* genomic and p53 protein status

TNBC subgroup					
	**TP53 genomic status**				
	wildtype	missensense	other	**total**	**p (chi square)**
**p53 protein status**					
wildtype pattern	13 (31.7%)	7 (17.1%)	21 (51.2%)	41	
overexpression	14 (12.7%)	88 (80.0%)	8 (7.3%)	110	
loss	15 (48.4%)	2 (6.5%)	14 (45.2%)	31	<0.0001
**total**	42	97	43	182	
